# Verruciform Acral-Associated (Vacas) Xanthoma: Case Report and Review

**DOI:** 10.7759/cureus.16569

**Published:** 2021-07-22

**Authors:** Tejas P Joshi, Antoanella Calame, Philip R Cohen

**Affiliations:** 1 Dermatology, Baylor College of Medicine, Houston, USA; 2 Dermatology/Dermatopathology, Compass Dermatopathology, San Diego, USA; 3 Dermatology, Scripps Memorial Hospital, La Jolla, USA; 4 Dermatology, San Diego Family Dermatology, National City, USA

**Keywords:** acral, foot, hand, sole, toe, vacas, verruca, verruciformis, wart, xanthoma

## Abstract

Verrucous xanthoma is a benign histiocytic lesion of macrophage derivation. We describe a woman with a non-mucosal verruciform xanthoma located on her right thumb and review the features of patients with verruciform acral-associated xanthoma. A 69-year-old woman presented with a lesion on her right thumb of eight years duration that had been previously treated with liquid nitrogen cryotherapy without resolution. An initial biopsy was consistent with the surface of a callous. A second biopsy demonstrated a verruciform xanthoma. The patient elected to apply lactic acid 12% twice daily and pare the lesion with a pumice stone once weekly; this resulted in flattening of the xanthoma-associated hyperkeratosis. Acral verruciform xanthoma has, albeit rarely, been described on the hands and feet of individuals. Including the patient in this report, six individuals have been reported with verrucous xanthoma on the hands and 12 individuals have been reported with a verrucous xanthoma on the feet. Verruciform xanthoma most commonly occurs on the oral mucosa. Genital lesions are also a frequent site. Acral-distributed verruciform xanthoma is rare; we propose that a verruciform xanthoma that occurs on acral sites be referred to as a verruciform acral-associated (Vacas) xanthoma.

## Introduction

Verruciform xanthoma is a benign, usually solitary, lesion. It typically occurs on the oral mucosa. Foamy histiocytes in the papillary dermis are the pathologic hallmark. The overlying epidermis frequently shows acanthosis and papillomatosis [1].

Extra-oral verruciform xanthomas have also been observed. The most common location is the penis and scrotum in men or the vulva in women. This variant has been referred to as a verruciform genital-associated (Vegas) xanthoma [2].

Albeit rarely, verruciform xanthoma can occur at other non-oral locations [3-20]. We describe a woman with a verruciform xanthoma on her right thumb and review the published reports of patients with verruciform xanthoma on their hands and feet. We propose that verruciform xanthomas at these acral sites be referred to as a verruciform acral-associated (Vacas) xanthoma.

## Case presentation

A 69-year-old Caucasian woman presented for evaluation of a chronic lesion on her right thumb. The thumb lesion initially appeared ten years ago; several of her prior clinicians had clinically diagnosed the lesion as a verruca vulgaris. It has been repeatedly treated with liquid nitrogen cryotherapy.

**Figure 1:**
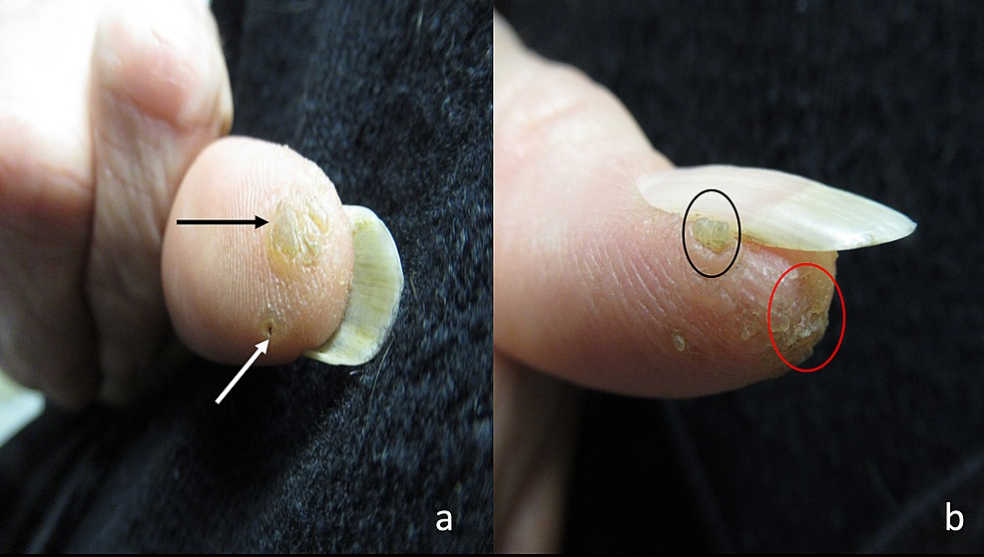
Clinical presentation of verrucous xanthoma on the distal right thumb. The palmar view (a) of the keratotic, verrucous plaque shows not only the site of the initial biopsy (white arrow pointing to purple dot), but also the site of the second, deeper biopsy (black arrow). The lateral view (b) shows both the verrucous plaque underneath the lateral nail fold (black circle) and the larger portion of the verrucous xanthoma on the tip of the thumb (red circle).

Cutaneous examination shows a verrucous 20 x 10 millimeter plaque on the palmar distal right thumb and lateral nail fold (Figure 1). The keratotic lesion had raised areas that measured 10 x 10 millimeters and 5 x 5 millimeters on the pad of the digit. The lateral nail fold also showed a keratotic plaque measuring 4 x 2 millimeters.

**Figure 2:**
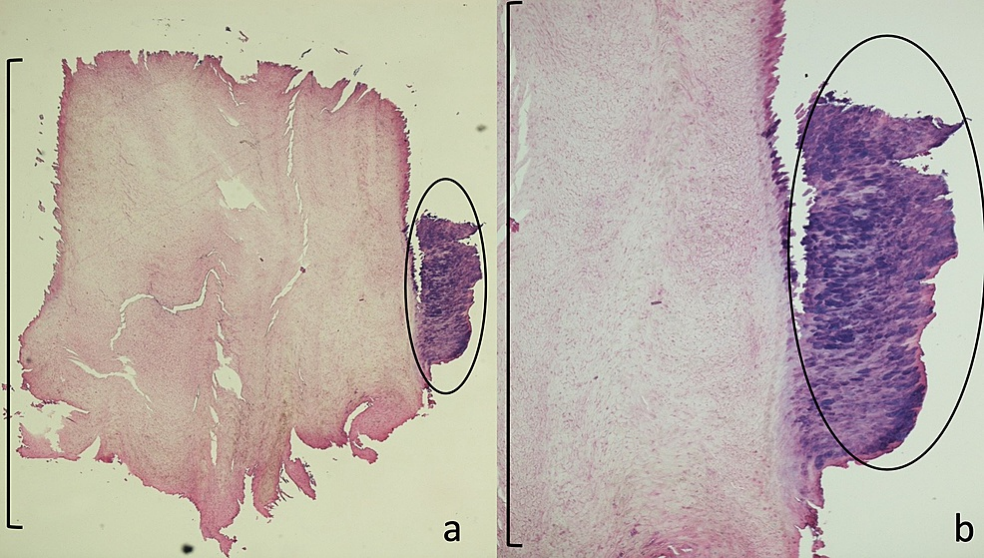
Histopathology of the initial biopsy of the superficial portion of verrucous xanthoma on the right thumb. Lower (a) and higher (b) magnification views of the stratum corneum overlying a verruciform xanthoma show massive compact orthokeratosis (within black bracket) and parakeratosis (within the black circle). These pathologic changes were initially interpreted as a clavus (hematoxylin and eosin: a, x2; b, x4).

A 2-millimeter biopsy using the punch technique was performed from the smaller portion of the ventral surface of the thumb. Microscopic examination showed marked orthokeratosis and parakeratosis; neither deeper layers of the epidermis nor the underlying dermis were seen (Figure 2). The dermatopathologist suggested a diagnosis of clavus.

**Figure 3:**
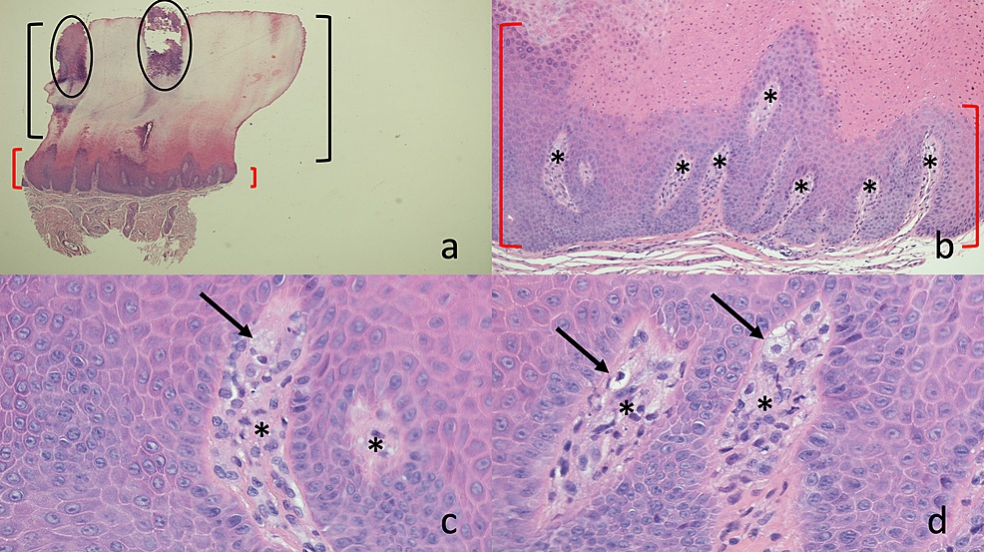
Histopathology of the biopsy of the larger, palmar portion of the verrucous xanthoma on the distal thumb. Lower (a and b) and higher (c and d) magnification views of the pathologic changes of a verruciform xanthoma on the tip of the right thumb of a 69-year-old woman. There is complete orthokeratosis (within black brackets) and focal mounds of parakeratosis (within black circles) in the stratum corneum (a and b). Thickening (acanthosis) and undulation (papillomatosis) of the epidermis (within the red brackets) is also present (a and b). The dermal papillae (black asterisks) extend upward into the epidermis (b, c, and d). In addition to lymphocytes, foamy histiocytes (black arrows) are also present (c and d) (hematoxylin and eosin: a, x2; b, x10; c, x40; d, x40).

A deeper and wider, 3-millimeter biopsy using the punch technique was performed of the larger, palmar portion of the lesion on the distal right thumb. Microscopic examination showed a hyperkeratotic cornified layer with columns of parakeratosis overlying areas of the epidermis; there was no granular cell layer. There was also acanthosis and papillomatosis with an upward extension of the dermal papillae into the lower portion of the epidermis. In addition to lymphocytes in the papillary dermis, there were foamy histiocytes (Figure 3).

These pathologic changes establish the diagnosis of a verruciform xanthoma. The benign diagnosis was discussed with the patient. She decided to apply lactic acid 12% cream twice daily to her thumb and weekly pare the residual lesion using a pumice stone. Flattening of the overlying hyperkeratosis was observed at a follow-up examination two months later.

## Discussion

The original paper on verruciform xanthoma is credited to Shafer and was described in 1971 [3]. Santa Cruz and Martin described the first two patients with extra-mucosal verruciform xanthoma on the vulva [4]. Subsequently, other extra-oral sites have also been observed; indeed, Vegas xanthomas refer to these lesions when they appear in the genital regions of men and women [2,5].

Verruciform xanthomas occurring in regions other than the oral and anogenital mucosa were reviewed by Blankenship et al. in 2013 [6]. The xanthomas were most commonly associated with conditions of chronic inflammation or trauma, as well as in the settings of chronic graft versus host disease, chronic lymphedema, and Congenital Hemidysplasia with Ichthyosiform nevus and Limb defects (CHILD) syndrome. The investigators observed that the presence of a verruciform xanthoma in the absence of any disease was a rare occurrence [6].

Acral verruciform xanthomas have been observed. They can be located on the hands (Table 1) [7-11].

**Table 1:** Characteristics of patients with verruciform xanthomas on the hand Abbreviations: #, number of lesions on the hand; 2, second or index finger; 3, third or long finger; 5, fifth or little finger; A, age (years); AA, African American; AD, associated disease; C, case; Ca, Caucasian; CHILD, Congenital hemidysplasia with ichthyosiform nevus and limb defects; cm, centimeter; CR, current report; Ery, erythematous; F, finger; fis, fissuring; H, hand; Hi, Hispanic; L, left; LA, lactic acid 12% topical cream; Lin, linear; LN2, cryotherapy with liquid nitrogen; NS, not stated; P, paring with pumice stone weekly; pap, papule; plq, plaque; R, right; Ra, race; Ref, reference; S, sex; synd, syndrome; T, thumb; Tx, treatment; Ver, verrucous; W, woman; yel, yellow; &, and. ^a^Lesions also present on the right labium majus and right upper inner thigh. ^b^Lesions also present in the right axillary region, right buttock, and some toes of the right foot. ^c^Lesions also present on the fourth and fifth digits of right foot. ^d^Lesions also present on the left toes and heel and the left genital area.

C	A, S, Ra	#	Site	Morphology	Tx	AD	Ref
1	3, W, Hi	3^a^	RH, RF	Elevated & ver	NS	CHILD synd	[7]
2	8, W, NS	6^b^	R2, R3, R5	Lin 4-5 cm horny masses	NS	CHILD synd	[8]
3	21, W, AA	3^c^	L3	Ver	LN2	None	[9]
4	27, W, NS	3^d^	LH	Ery plq & fis	NS	CHILD synd	[10]
5	69, W, Ca	1	RT	Ver, keratotic plq	LA, P	None	CR
6	80, W, NS	1	LT	Ill-demarcated yel tan 0.8 x 0.8 cm pap	LN2	None	[11]

Acral verruciform xanthoma can also be located on the feet (Table 2) [8-10,12-19]. 

**Table 2:** Characteristics of patients with verruciform xanthomas appearing on the feet Abbreviations: #, number of lesions on the hand; 2, second toe; 3, third toe; 4, fourth toe; 5, fifth toe; A, age (years); AA, African American; AD, associated disease; Ann, annular; C, case; Ca, Caucasian; CHILD, Congenital hemidysplasia with ichthyosiform nevus and limb defects; cm, centimeter; Cs, corticosteroids; CS, compression stocking wearing; CP, compression pump use; C&D, curettage and electrodessication; D, dorsum; D3, vitamin D3 analog; D&E, debridement and excision; Ery, erythematous; exo, exophytic; F, foot; fis, fissured; fun, fungating; Gt, great toe; H, heel; Hem, hemorrhagic; Hk, hyperkeratotic; Inf, infiltrated; ILVEN, inflammatory linear verrucous epidermal nevus; L, left; Lat, lateral; LCS, leaky capillary syndrome; LD, lymphedema; lin, linear; LP, lymphedema praecox; M, man; MD, Milroy disease; nod, nodule; NS, not stated; NT, no treatment; pap, papules; ped, pedunculated; plq, plaque; R, right; Ra, race; Ref, reference; S, sex; SC, skin-colored; SE, surgical excision; sm, smooth; synd, syndrome; T, toe; Tx, treatment; Ver, verrucous; W, woman; yel, yellow; &, and. ^a^Lesions also present in the right axillary region, right buttock, and three fingers of right hand. ^b^Lesions also present over left calf and left lower leg. ^c^Lesions also present on right trunk and right arm and leg. ^d^Lesions also present over lateral aspect of right leg. ^e^Lesions also present over the right inguinal fold, gluteal fold, and leg. ^f^An additional lesion present on the left third digit. ^g^Lesions also present on left genital area and left side of hand.

C	A, S, Ra	#	Site	Morphology	Tx	AD	Ref
1	8, W, NS	6^a^	RFT	Lin 4-5 cm horny masses	NS	CHILD synd	[8]
2	10, M, NS	3^b^	LDF, LDT	Inf yel pap & plq	NS	LP	[12]
3	10, W, Ca	2	LGt, L2, L3, L Lat F, L Lat H	Ann or lin yel-white ver pap	CS, CP	LD	[13]
4	12, W, Ca	2	RDGt, RD2, LGt	SC ver & hem pap	C&D	LCS	[14]
5	15, W, NS	3^c^	RF	Large, ery fleshy tumor	Cs, D3	CHILD synd	[15]
6	17, W, NS	2^d^	LDF	Ver & exo plq	SE	ILVEN	[16]
7	17, W, NS	4^e^	RF	Ver ery plq	D&E	CHILD synd	[17]
8	18, M, Ca	1	RDGt, RD2, RD3	Ery yel ped & sm-topped pap & plq	C&D	MD	[14]
9	21, W, AA	3^f^	R4, R5	Hk pap & lin plq	NS	None	[9]
10	27, M, Ca	1	LGt	1 cm yel ver nod	NS	LD	[18]
11	27, W, NS	3^g^	LH, LT	Ery lin & fis plq	NS	CHILD synd	[10]
12	36, W, AA	1	RGt	Yel fun ver nod	NT	LD	[19]

Verruciform xanthoma of the hand was observed in six patients, including our patient. All of the patients were women. The women ranged in age from three years to 80 years (median, 24 years) at diagnosis.

The hand lesions were located most commonly on the thumb (two patients) and the middle finger (two patients). One patient also had a verrucous xanthoma on her index finger. Verrucous xanthomas arising in the setting of CHILD syndrome in three of the patients had almost complete involvement of the hand. Four of the women also had more than one additional verrucous xanthoma that was not located on the upper extremity.

Verrucous xanthoma of the feet was observed in 12 patients. Nine of these patients were women and two were men. The women aged in range from eight years to 36 years (median, 17 years) at diagnosis. The men aged from ten years to 27 years (median, 18 years).

The feet lesions were located most commonly on the toes. In the setting of CHILD syndrome, most of the patients’ xanthomas involved almost the entire foot. Seven of the patients also had at least one additional verrucous xanthoma that was not located on their feet.

The pathology of acral verruciform xanthomas is similar to that of verruciform xanthomas observed in mucosal and other non-mucosal locations [1]. The epidermis most commonly shows hyperkeratosis (orthokeratosis or parakeratosis or both) and acanthosis with papillomatosis [7,9,11-19]. Other epidermal features, less frequently observed, have included psoriasiform hyperplasia, hypergranulosis, and elongated rete ridges [7,9,13,15,16,18,19]. In the dermal papillae and superficial papillary dermis, foamy histocytes are present [7,9,11-19]. Other findings that have been described in the dermis were angiogenesis or edema and an inflammatory infiltrate of one or more of the following cells: lymphocytes, neutrophils, or plasma cells [9,11,15,18].

Acral verruciform xanthomas can be identified on hematoxylin and eosin staining. Moreover, periodic acid-Schiff staining can also be used to more readily identify the foamy cells [11,18]. In addition, the histiocytes can also be labeled using an immunoperoxidase stain such as cluster of differentiation 68 (CD68) that stains macrophages [1]. 

Acral verruciform xanthomas, predominantly those of the lower extremity, may be associated with CHILD syndrome and lymphedema (Table 2) [8-10,12-19]. One patient with a verruciform xanthoma appearing on the feet also had concomitant leaky capillary syndrome [14].

Three of the six patients who had a verrucous xanthoma on the hand and four of the twelve patients who had a verrucous xanthoma on the foot also had CHILD syndrome, a rare, X-linked dominant condition. The cutaneous manifestation of CHILD syndrome is characterized by an inflammatory ichthyosiform nevus that lateralizes to one side. Although verrucous xanthoma is currently considered to be an unusual finding in CHILD syndrome, several researchers have concluded that it is a characteristic associated with the disease [17].

We also found that five of the 12 patients who had verrucous xanthoma on the foot also had lymphedema. The precise association between lymphedema and verruciform xanthoma remains to be elucidated. However, Hunter et al. have speculated that hypoplastic or incompetent lymphatics that are present in the setting of lymphedema can permit lipoprotein escape from the lymphatics; subsequent phagocytosis of the lipoprotein may contribute to verruciform xanthoma formation [20].

Wu and Wagner also reported a 12-year-old boy who had verruciform xanthoma in the setting of a leaky capillary syndrome, a state of hemodynamic disarray precipitated by vasoactive substances that result in an increase in vascular permeability. The xanthomas presented as skin-colored, verrucous and hemorrhagic papules on both of his great toes and his right second toe. The investigators proposed that the pathogenesis of verruciform xanthoma in the setting of the leaky capillary syndrome is similar to the development of verruciform xanthoma in the context of lymphedema [14].

Acral verruciform xanthoma is a benign lesion. After a biopsy, the individual lesion can be observed. Alternatively, the xanthoma can be excised with scalpel or laser surgery. The pathogenesis of verruciform xanthoma remains to be established.

The acronym Vegas xanthoma is the established nomenclature to describe verruciform xanthoma in the genital region. Therefore, using a similar manner of nomenclature, we suggest that a verruciform xanthoma located on the distal extremities be referred to as a Vacas (*v*erruciform *ac*ral-*as*sociated) xanthoma. The 'V' is the first letter of verruciform, the 'ac' are the first two letters of acral, and the 'as' are the first two letters of associated. Our proposed acronym incorporates the Spanish word vacas for which the English translation is cows.

## Conclusions

Verruciform xanthoma is typically observed in the mouth. Non-oral verruciform xanthoma is commonly located in the genital region and referred to as a Vegas xanthoma. Albeit less frequently, as in the reported woman with a verruciform xanthoma on her right thumb, verruciform xanthomas may develop on the distal extremities. Verruciform xanthomas appearing on acral locations have been associated with CHILD syndrome and lymphedema. The acronym Vacas (verruciform acral-associated) xanthoma has been proposed when referring to verruciform xanthomas that occur on the distal extremities.
